# The Use of Suprarenal in Dental Cases

**Published:** 1902-06

**Authors:** 


### Reviews of Dental Literature.

---

The Use of Suprarenal in Dental Cases. By E. A. Peters, M.D. (Cantab.), M.R.C.S., L.R.C.P. (Lond.).

Mr. President and Gentlemen,—Thanks to the courtesy of the President, I am privileged to address you to-night, and I do so more with the expectation of eliciting the results of your experience and suggesting the trial of a method which has been successful in my hands, than to attempt to lay down rules for the treatment of a large class of cases which need daily relief from all sections of the healing profession.

In dentistry there seems to me to be three groups of cases in which suprarenal may be more generally employed: (1) Where it may be used as a hæmostatic; (2) as an adjuvant to local anæsthesia; (3) as a local application to allay the pain of inflammation.

For such purposes a liquid extract of the gland is required. The best results are obtained by employing the extract of the fresh gland, but for obvious reasons the use of such a preparation is unpractical; and I have employed for most of my cases an extract, freshly prepared each day of employ, from the tabloids of dried gland sold by Messrs. Burroughs and Wellcome. The tabloids are powdered and shaken with cold water, so as to form ten or twenty per cent. mixtures according to the weight of the originally fresh gland. The containing vessel is then placed in boiling water and heated with occasional shaking for ten minutes. The filtrate is secured, and is reliable for two days or so. If allowed to stand, a dark-brown precipitate forms and carries with it the efficient properties. A most fugitive and shy body is the active constituent, and some of the preparations on the market are singularly deficient in giving the typical physiological reaction of the extract. The vasoconstricting body is said to be an alkaloid.

A one-fourth per cent. solution of suprarenal extracted from the tabloid and diluted with normal saline will give the typical suprarenal reaction when five minims are injected beneath the skin of the forearm. In five to ten minutes a white patch appears over the site of injection and the hair papillæ stand out, forming a goose skin. In marked cases the skin takes on the appearance of rough mosaic. This area has slightly diminished sensibility, and when pricked does not bleed as readily as usual. In this test we have a convenient method for comparing the different strengths of preparations of suprarenal.

If suprarenal extract as a ten or twenty per cent. solution is packed on pledgets of wool into the nose for twenty minutes, the mucous membrane absorbs part of the extract; it then shrinks and becomes white. In some noses it is necessary, on account of this great shrinkage, to repack the nose for another twenty minutes. When the pledgets of wool are removed portions of the mucous membrane or bone can be removed without a drop of blood falling from the nose. Bleeding sets in at the end of two hours, but the operator is prepared for this and has plugged the nose. Occasionally the bleeding is only modified in amount.

Suprarenal solutions in large amounts have been injected into the subcutaneous tissues of rabbits; a ten per cent. solution prepared as above was used for this purpose. When a little over one per thousand of suprarenal, as compared with weight of rabbit, is employed and daily injected, the rectal temperature falls. At two per thousand the animal loses body weight; when five per thousand is reached the hair over the area falls out and gangrene appears in the area of injection. All this is apparently due to the vasoconstriction, and is on a parallel with the ergot gangrene of our forefathers. If the same ratio of body weight holds good in man, then it would be necessary to inject rather over sixteen ounces of the ten per cent. solution before serious symptoms set in. It is rarely needful in practice to use more than one drachm. So you will see that this extract can be safely used in practice.

The principal action of the extract is to effect a contraction of the involuntary muscles of the arterioles, producing a local ischæmia. According to some there is also a central action. If one suprarenal gland is removed the other hypertrophies (Moore, Vincent, Peters).

1. The hæmostatic action of suprarenal was soon turned to clinical use when Oliver had demonstrated extracts of the gland to have such a powerful vasoconstrictor action, and for this purpose it has been applied in nearly every part of the body. In dental surgery it is useful in stopping hemorrhage from a septic socket, or elsewhere. It has been said that suprarenal extract will check the bleeding of hæmophilia, but of this I have no experience. Possibly some of those present could give their experience on this point.

To obtain the hæmostatic effect ten per cent. suprarenal or a stronger preparation should be applied on a pledget of wool with sustained pressure.

2. Suprarenal is also useful as an adjuvant in producing local anæsthesia. As previously pointed out, the sensory perceptions of the skin over the site of a hypodermic injection of suprarenal are merely dulled—there is no absolute analgesia or anæsthesia. But if cocaine or eucaine is added, the ordinary anæsthesia pertaining to injections of these drugs is prolonged, and a smaller amount of these drugs is required when thus combined with suprarenal to cause an equal depth of anæsthesia. Besides this, cocaine so employed is less poisonous, for it is retained in the tissues for a longer time. Cocaine is a vasoconstrictor when given in small doses; in larger quantities it acts as a vasodilator.

If a dilute solution of cocaine is placed on the web of a frog the arterioles are seen to shrink up, but dilate at the end of three to five minutes. If another diluted drop is placed on the web similar phenomena recur. If, however, a crystal of the cocaine salt is directly applied, then instant dilation obtains. Cocaine, according to Mosso (*Archives Ital. de Bolog.,* Turin, 1890, 8, 323), is excreted unchanged through the kidneys. In the ordinary method of application or injection the soluble salt is swept out of the tissues to the central nervous system and there produces its disastrous paralyzant effects. In the case of the frog's web this takes place within two minutes. I have collected a list of published cases of cocaine poisoning. In this schedule it appears that concentrated solutions introduced into an empty stomach in an attempt to inject a quantity into the dense mucous membrane, or accidental swallowing, account for many of the cases. Cocaine, unlike eucaine, forms no local compound. Reclus (*Le Cocaine en Chirurgie,* Paris, 1895) limits the internal use of cocaine to 1.1 grains, but it is usual in throat work to use pledgets of wool soaked in ten per cent. cocaine, and often there must be five or more grains in the nose. Now, poisoning symptoms are almost unknown in throat work, though a rapid pulse, one of the early symptoms, is frequently recognized. I have worked for years in a throat hospital, but have never seen a case of definite poisoning, so safe is the pledget system.

Suprarenal is of real advantage by contracting the vessels, it localizes the drug, and produces a more prolonged anæsthetic effect, while the drug is more slowly absorbed by the vascular system.

Eucaine, on the other hand, is a vasodilator, but though a powerful paralyzant it forms a local compound with the local tissues, and so the central nervous system is remote to its sphere of influence (Peters, M.D., Thesis, Camb., 1900). Even the anæsthesia of eucaine is made deeper and more prolonged by its combination with suprarenal. In ordinary doses suprarenal overcomes the vasodilation due to eucaine.

There is another advantage which the combined anæsthetic has: it is to render inflamed tissues anæsthetic, a condition which ordinary cocaine fails to bring about for some unexplained reason. Pledget application of a solution containing five per cent. cocaine and ten per cent. suprarenal may be carried out for half an inch each side of the tooth, followed by the injection of five minims of the twice diluted saline solution in the region of the infra-orbital or mental nerve as required. It is left for absorption to delete the deeper nerves; but it would be possible, if necessary, by a suitable injection into the region of the spine of Spix, or into the maxillary antrum, to cut out the trunks of the deep nerves. I have never seen this done; perhaps some one here has had some experience of this method.

Corning, an American, was the first to point out that an injection of cocaine in the region of a nerve-trunk resulted in the temporary deletion of the functions of the nerve. By applying his method I was able to map out the cutaneous supply of certain nerves, and to show that on the forearm the twigs of one nerve overlapped the other by half an inch (M. D. Thesis, Camb., 1900).

Of course, this method is open to the same drawback as all local methods,—*i.e.*, the patient is conscious of an operation in progress; the minutes of operative procedure are not blotted out for him as in the case of a general anæsthetic. In my own department local anæsthesia is decidedly satisfactory, and a patient who has once undergone a nasal operation under such local conditions will rarely call for any other method if undergoing a subsequent operation.

3. Last of all, we come to the third use of suprarenal extract,—*i.e.*, its application alone or in combination with one-half per cent. of cocaine hydrochloride for the relief of inflammatory pain. I wish to draw your particular attention to such use.

I have described elsewhere (*Lancet*, March 2, 1901) cases where the pain and soreness of laryngeal tuberculosis, recurrent cancer of the breast, of pain which is essentially due to ulceration and chronic inflammation, were relieved by such treatment. In most chronic inflammations there are periods of acute inflammation. Besides these I have employed the extract in dental cases to tide patients over a painful period before they could come into the hands of the dental surgeon. The extract was applied as described above,—*i.e.*, a pledget of wool soaked in ten or twenty per cent. suprarenal extract is tucked up between the cheek and the gum, and the solution painted on the palatal aspect if the patient cannot allow a similar pledget to cling to the palatal surface for a short time; the pledget should reach half an inch on each side of the tooth. The labial pack should be allowed to remain during sleep, as otherwise the pain may be experienced at the end of two or more hours (*cf.*, the hemorrhage which follows the extract when used in nasal operations after the lapse of two hours).

I will submit two cases to your notice.

1. A lady two days after an operation had been performed complained of great pain which had kept her awake the previous night. I detected a swelling on the palatal side of the alveolus in connection with one of the molar teeth. Ten per cent. extract was applied in the way I have described; she slept that night, and said the pain was rendered bearable. Three days later a small alveolar abscess opened without surgical interference.

2. In the second case pain and tenderness was experienced on the palatal surface corresponding to the palatal root of the first molar, where a slight swelling could be detected. Cocaine, chloroform, iodine, and aconite were all used, and carbolic acid was packed up in between the dental interval, all to no purpose. Applications of the extract, however, tided the patient over for four days, when the pain subsided. About a year later considerable pain was experienced in the same tooth and the surrounding maxilla. On the sixth day of pain Mr. Maggs removed the tooth, finding it septic and an abscess at the apex of the palatal root.

I may add that I have packed the dental intervals with wool soaked in one in ten carbolic acid or five per cent. cocaine dissolved in beechwood creosote. The relief of pain by this method is probably due to vasoconstriction. The arterioles supplying the area contracted and the distention by blood and exuded serum diminished. The increased pain of inflamed dense tissues is said to be due to increased pressure and traction of nerves. Pain by some is said to obtain when a nerve of common sensibility is affected; others are of opinion that there are specific nerves for pain. It is interesting to note that in inflammation of the skin and subcutaneous tissues all the specific functions are disordered, giving abnormal sensations of common sensibility—heat or cold, weight and pain. The action of suprarenal is to render dull all these sensations, and not to completely delete them. At this point it is necessary to ask the question, Does suprarenal influence the final results of inflammation in an unfavorable way? Personally, I have not seen any necrosis or symptoms of general infection follow its use in the manner specified; the mucous membrane is not damaged. Further, such treatment is in accordance with the employment of depletion and cold applications which are usually accepted for the treatment of acute inflammation. The serious symptoms of inflammations of bone and other dense or imprisoned tissues are caused by the strangulation of vessels by excessive exudation. Possibly, then, suprarenal reduces the ultimate damage. The relief to the patient is considerable; he is able to work and to sleep under its local influence. I do not say that the patient receives as much relief as he obtains from a dose of morphia, which acts on the central nervous system, but suprarenal has the advantage of being innocuous when employed in the manner detailed, and in spite of its instability the use of the extract, prepared as recommended, will give fairly constant results.—*Transactions of the Odontological Society of Great Britain.*

